# Quest for 2,3-Secopyramidane: Computations Hint at Elusive Structure during Skattebo̷l Rearrangement of Vinylcyclopropylidene

**DOI:** 10.1021/acs.joc.5c03248

**Published:** 2026-04-15

**Authors:** Murray G. Rosenberg, Udo H. Brinker

**Affiliations:** † Independent Researcher, 216 Harry L Dr., Johnson City, New York 13790, United States; ‡ Institute of Organic Chemistry, 27258 University of Vienna, Währinger Str. 38, Vienna 1090, Austria

## Abstract

The ωB97X-D/def2-TZVP + 0.955­(*E*_ZPV_) theoretical model was used to determine if 2,3-secopyramidane (tricyclo­[2.1.0.0^1,3^]­pentane) participates during the Skattebo̷l rearrangement of *endo*-2-ethenylcycloprop-1-ylidene. Intrinsic reaction coordinate animations show an almost barrierless attempt at 1,2-addition of the divalent C atom to the ethenyl double bond because the transition state is stabilized by intramolecular σ- and π-complexation. The formation of 2,3-secopyramidane is incomplete, however, and retro-1,2-addition takes over, leading to ring-puckered cyclopent-3-en-1-ylidene, a *bis*homoaromatic carbene. Ring-flattening is followed by a 1,2-H atom shift yielding cyclopenta-1,3-diene.

## Graphical abstract

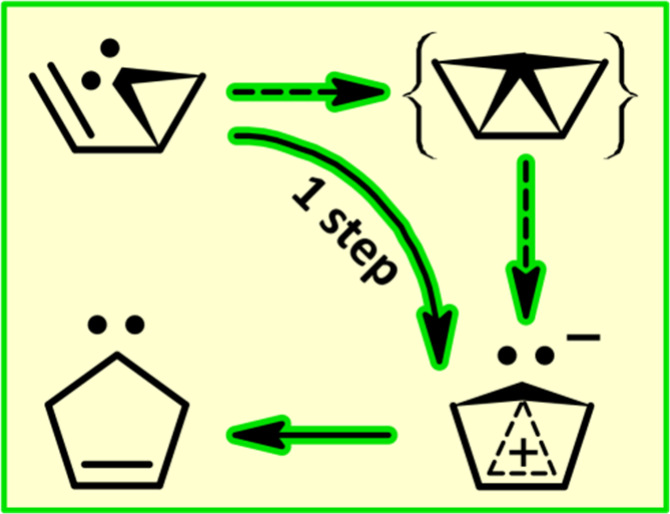

## Introduction

The eye-catching dandy 2,3-secopyramidane (**1**; tricyclo­[2.1.0.0^1,3^]­pentane)^1^ is a conceptual hydrogenation product of pyramidane (**2**; tetracyclo­[2.1.0.0^1,3^.0^2,5^]­pentane)^2–6^ (Scheme 1, path a; Scheme S1), another elusive gem whose mystique harkens back to Earth’s ancient megalithic pyramids. Regrettably, direct observation of highly strained **1** and **2** has been challenging because they are expected to be highly reactive molecules.^1^ Although computations support the kinetic stability of *tetra*cyclic **2**,^2–6^ results for *tri*cyclic **1** are less conclusive.^1^ Preparative methods for tricyclo­[2.1.0.0^1,3^]­pentanes are shown in Scheme 1. Ring-closure may be achieved with **3** via a metal–halogen exchange and backside displacement (Scheme 1, path b)^1^ or with **4** via a carbene 1,2-addition (Scheme 1, path c).^7–12^ Computational and experimental evidence has been offered claiming path b generates *trans*-tricyclo­[2.1.0.0^1,3^]­pentane (**5**)—a stereoisomer of **1** (Scheme 1). According to the MP2/6-31G­(d) theoretical model, **5** has a strain energy of 143 kcal/mol.^1^ Empirical evidence for **5** comes from ^13^C NMR spectroscopy at *T* = –55 °C, which revealed a signal at δ_C_ = 38.8 ppm attributed to the 2 CH_2_-groups of **5**.^1^ The *trans* configuration was posited based on a coupling constant *J*_HH_ = 3.7 Hz. It is believed that *trans* isomer **5** generates cyclopent-3-en-1-ylidene (**6**), via a radical process,^1^ ultimately yielding cyclopenta-1,3-diene (**7**) via a 1,2-H atom shift. Path c (Scheme 1) proceeds via the carbene 2-ethenylcycloprop-1-ylidene (**8**) (Scheme 2).^7,8^ This cyclopropylidene derivative rearranges via a *bis*homologous ring-expansion to give the ring-puckered nonclassical carbene cyclopent-3-en-1-ylidene (**9**), which flattens to form classical carbene **6**.^9b,9c^

**Scheme 1:**
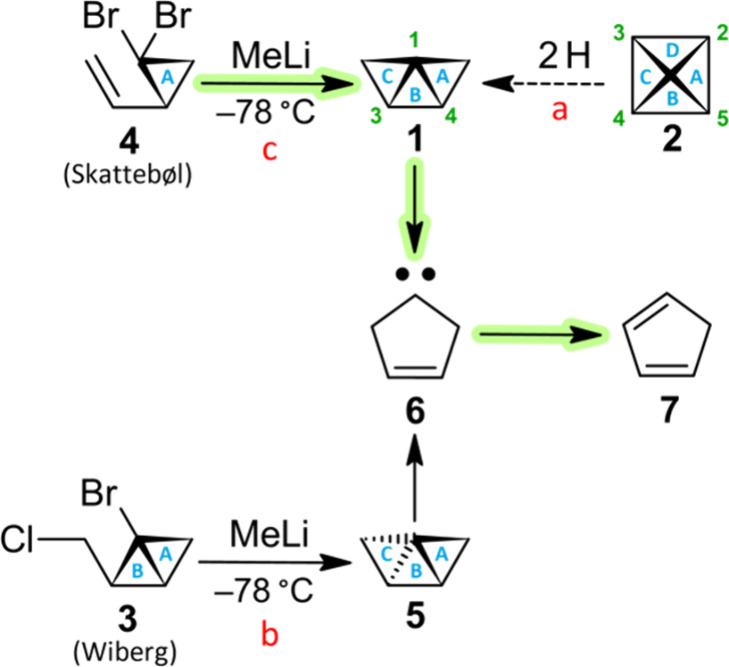
Strategies toward 2,3-Secopyramidane (**1**)

**Scheme 2:**
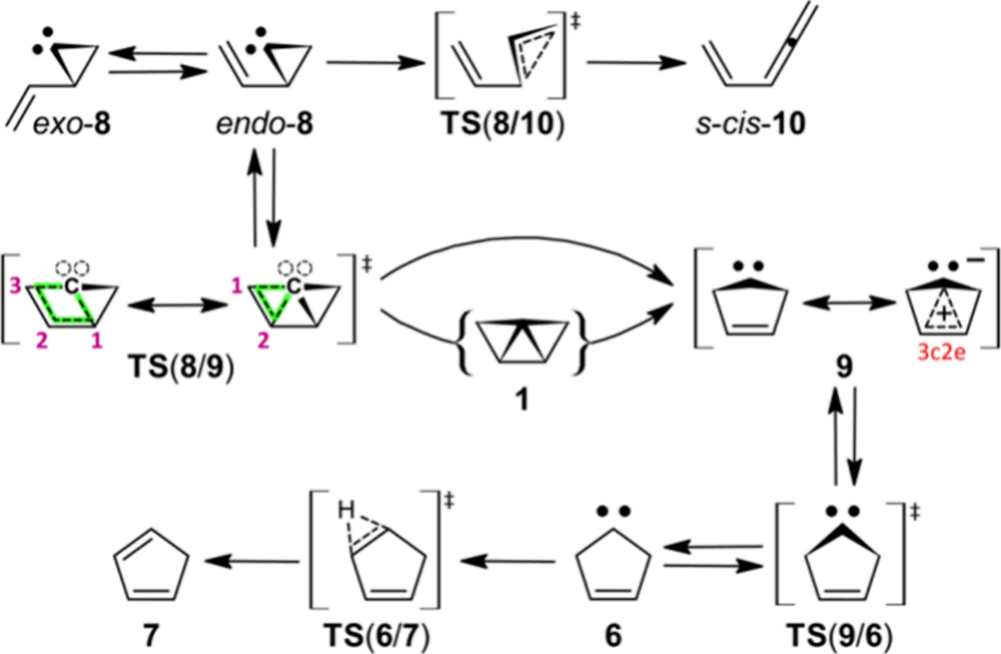
Fate of *endo*-2-Ethenylcycloprop-1-ylidene (*endo*-**8**)

The *Skattebo̷l rearrangement* **8** → **6** resembles a 1,3-C atom shift,^9b,9c,13^ but carbene **8** might initially form **1** via an intramolecular 1,2-addition generating two σ bonds from a π bond and electron lone-pair (Scheme 2).^13^ Indeed, forming cyclopropanes is a specialty of carbenes.^14^ Ring-expansion *endo*-**8** → **9** has been modeled computationally using MINDO/3 semiempirical calculations as well as the CCSD­(T)­(fc)/6-311+G­(3df,2p)//B3LYP/6-31G­(d) + 0.9806­(*E*_ZPV_) theoretical model.^15,16^ Both models indicate the presence of a π-complex within *endo*-**8**, wherein the occupied π-MO localized on the C–C double bond donates electron density to the divalent C atom’s unoccupied p orbital.^15,16^ Also, they affirmed ring-puckered conformer **9**, which lends credence to *endo*-**8** → **9** but does not disprove *endo*-**8** → {**1**} → **9** (Scheme 2). Note that penta-1,2,4-triene (**10**) is a side-product of carbene **8**. The production of allene **10** increases as reaction conditions rise above *T* = –78 °C.^17^

This inquiry seeks to uncover the role—*if any*—played by hypothetical structure **1**. To do so, hundreds of structures on the C_5_H_6_ potential energy surface (PES) in the vicinity of *endo*-**8** → **9** will be generated by performing a 2D grid calculation computed at the ωB97X-D/def2-TZVP level of theory (see Experimental Section).^18,19^ The inclusion of dispersion, use of a triple-ζ non-Pople basis set, and a large DFT integration grid should provide more accurate structures than those reported at the B3LYP/6-31G­(d) level of theory.^16^ The PES will be examined to locate **1** and determine whether the point represents an equilibrium geometry, a transition state (TS), or neither (e.g., a nonstationary point).

## Results and Discussion

Participation of **1** during the Skattebo̷l rearrangement (i.e., *endo*-**8** → {**1**} → **9** → **6**, Scheme 2) cannot be ruled out *a priori*. Molecular mechanics (DLFF3) gives a structure for **1** with an acute Cc–Cb–Ca–Ce dihedral angle (*ω*) of 70.2 deg (Figure S8e, Table S4). DFT computations were performed using the ωB97X-D/def2-TZVP + 0.955­(*E*_ZPV_) theoretical model (Figure 1). Elementary step *endo*-**8** → **9** was assessed by computing an intrinsic reaction coordinate (IRC) for **TS**(**8**/**9**) and animating it to observe the timing of bond breakage and formation (Scheme S2, Figure S2, Videos S1–S3). In addition to the IRC for the second step **9** → **6** (Scheme S3, Figure S3, Video S4), energy profiles were computed via a dihedral-angle step calculation (Δ*ω* = 2.5 deg; Scheme S4, Figure S4, Videos S5–S7). Forming **1** by compressing the Cc–Cb–Ca–Ce dihedral angle (*ω*) of **9** from 95.6 deg to 70.2 deg (*vide supra*) is “foiled” because the energy required rises sharply, according to the DFT method. Remarkably, the molecular-mechanics structure of **1** represents a TS ( ${\mathrm{\nu ̅}}_{\mathrm{TS}}$ = 335*i* cm^–1^), according to DFT frequency calculations. Animation of the corresponding normal mode revealed alternating twists of the CH_2_-groups and suggests **1** might be a racemization TS between *endo*-**8** and *ent*-*endo*-**8**. This hypothesis could not be verified, however, because an IRC could not be obtained for **1**. The molecular-mechanics structure of **1** transforms into carbene **9** when optimized as a ground state and into **TS**(**9**/**6**) when optimized as a transition state, according to DFT calculations.

**Figure 1:**
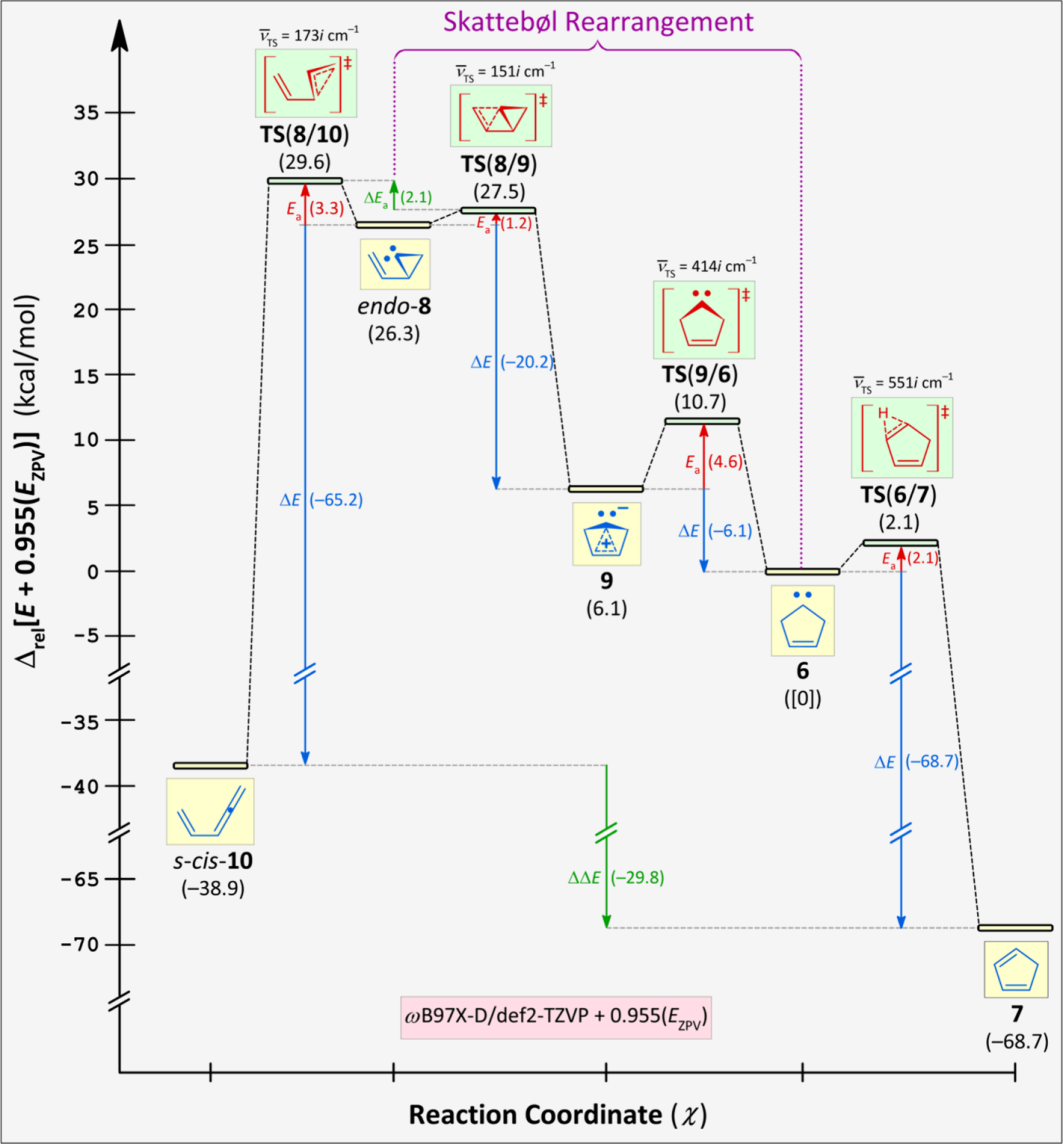
Reaction-step diagram showing *s*-*cis*-**10** ← *endo*-**8** → **9** → **6** → **7** (Δ_rel_ *E*(**6**) = [0] kcal/mol).

Significant ring-puckering within carbene **9** stems from delocalized 3-center 2-electron (3c2e) bonding, which comprises the occupied π-MO of the C–C double bond and unoccupied p orbital of the divalent C atom (Figure S1). Consequently, **9** features cyclopropenylium-like aromatic stabilization and is *bis*homoaromatic because the 5-membered carbocycle encompasses two nonparticipating CH_2_-groups.^20–22^ Despite this stabilization, **9** flattens to conformer **6** (Scheme S3, Figure S3, Video S4), which ultimately yields cyclic diene **7** (Scheme S5, Figure S5, Video S8).

The frontier MOs and energy levels for *endo*-**8**, **TS**(**8**/**9**), and **9** are presented in Table S2. Development of the 3c2e bond within **9** is manifested in the HOMO{−1}. The transient species *endo*-**8** and **TS**(**8**/**9**) have similar characteristics. Two intramolecular complexes exist within *endo*-**8** due to two types of intramolecular donor → acceptor FMO interactions (Figure 2): (a) the occupied Walsh orbital of the cyclopropylidene unit of *endo*-**8** overlaps with the vacant π^*^-MO of the ethenyl group, constituting a σ-complex (Δ*E* = 11.4 eV), while (b) the occupied π-MO of the ethenyl group of *endo*-**8** overlaps with the unoccupied p orbital of the divalent C atom, establishing a π-complex (Δ*E* = 8.9 eV). The energy-gap difference (ΔΔ*E*) of 2.5 eV signifies that the π-complex is more stabilizing than the σ-complex during *endo*-**8** → **TS**(**8**/**9**).

**Figure 2:**
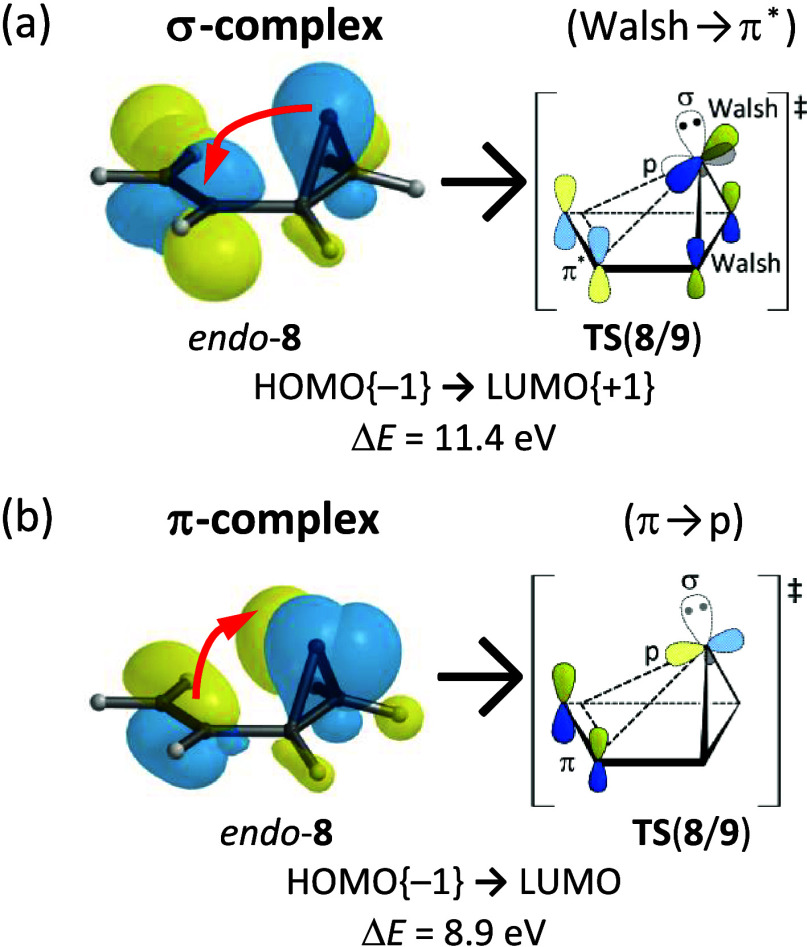
Two stabilizing intramolecular donor → acceptor MO interactions within **TS**(**8**/**9**) feature a (a) σ-complex comprising an occupied Walsh orbital and a vacant π^*^-MO and (b) π-complex comprising an occupied π-MO and a vacant p orbital.

Although the Lewis structure of **1** features an inverted 4°-C atom,^2,23^ its high-lying σ-MO is actually directed away from the other four C atoms. A similar σ-MO is prominent in **8** and **TS**(**8**/**9**). The Walsh orbital (HOMO{−1}) on the divalent C atom participates during the Skattebo̷l rearrangement but its electron lone-pair (HOMO) remains intact.^9c,15^ This is supported by isotopic labeling studies, which indicate that the divalent C atom is nonmigratory and retains its identity throughout the carbene–carbene rearrangement.^24,25^

As a consequence of its angle-strained geometry, cyclopropylidene (**11**) itself has a reported singlet–triplet energy gap (Δ*E*_S–T_) of –16.8 kcal/mol.^26^ The (σ^2^p^0^) electron configuration dominates over others, such as (σ^1^p^1^), because the σ-MO has a high degree of s-character. Nevertheless, the diradicaloid character of carbene **11** has been reported.^17b,27^ Thus, the triplet state of *endo*-2-ethenylcycloprop-1-ylidene (^3^ **8**) was computed using the UωB97X-D broken-symmetry DFT method and compared to **11**. The **:**CH_2_-corrected Δ*E*_S–T_ for **11** (−17.2 kcal/mol) is in good agreement with the G3­(MP2) method.^26^ The one for *endo*-**8** (−13.3 kcal/mol) is misleading, however. The equilibrium geometry of ^3^ **8** is congruent with that of triplet bicyclo[2.1.0]­pentane-1,3-diyl (^3^ **12**; Scheme 3). The terminal CH_2_-group of the ethenyl pendant of *endo*-**8** twists and bonds with the divalent C atom in the excited state.

**Scheme 3:**
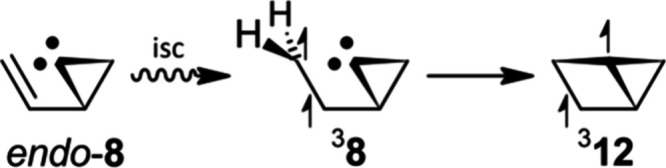
Hypothetical Formation of Bicyclo[2.1.0]­pentane-1,3-diyl (**12**)

Rotamerization within carbene **8** was modeled using a semirigid step calculation (Δ*ω* = 5 deg; Scheme S7, Figure S7, Video S9). The results show a ca. 1-kcal/mol preference for *endo*-**8** over *exo*-**8** due to intramolecular donor → acceptor complexation, which appears to outweigh increased steric congestion. Thus, *endo*-**8** is poised to undergo rapid intramolecular 1,2-addition with an activation energy (*E*_a_) of just 1.2 kcal/mol (with scaled ZPV-correction). The following ZPV-corrected thermodynamic values (Table S5) were computed for *endo*-**8** → **9** (Scheme 2) at *T* = 25 °C (298.15 K): activation enthalpy (Δ*H* ^‡^) = 1.1 kcal/mol, activation entropy (Δ*S* ^‡^) = –1.1 (cal/mol)/K, and activation Gibbs free-energy (Δ*G* ^‡^) = 1.4 kcal/mol. Ring-closure to a cyclopropane has a negative Δ*S* ^‡^. This ensures that Δ*G* ^‡^ is positive and that cyclopropanation within *endo*-**8** decreases with increasing *T*. Thus, the amount of allene **10** increases at temperatures above *T* = –78 °C (Scheme S6, Figure S6, Videos S10, S11).^9,17^

Bond breakage and formation during *endo*-**8** → **9** (Figure 3, red curve) can be simulated by varying 2 distances (*r*) within the carbene (Figure 3a, Figure S8a). So, a grid calculation (Table S3) was performed using *r*(Ca–Ce) and *r*(Ca–Cc) (Table S4). Relative energies are rendered as a 2D contour plot (Figure 3b) and a 3D potential energy surface (Figure 3c). Three mechanistic pathways are illustrated in the corresponding More O’Ferrall–Jencks diagram (Figure 3a):^28^ (1) dissociative asynchronous (turquoise dashed curve), (2) associative asynchronous (red dashed curve), and (3) synchronous (black dashed line). The synchronous pathway is the most expedient route but not the lowest in energy. It represents a 1,3-C atom shift. The dissociative pathway is unlikely because it traverses a higher-energy region. The associative pathway is most likely because it traces the lowest-energy route. The actual IRC path (dashed red curve) of the key elementary step was plotted in Figure 3b using the *r*(Ca–Ce) and *r*(Ca–Cc) values of each IRC structure. Its course most closely matches the associative pathway of Figure 3a. Three IRC animations for *endo*-**8** → **TS**(**8**/**9**) → **9** (Videos S1–S3) were prepared for inspection. The first one emphasizes the attempted asymmetric 1,2-addition component of the elementary step while the second highlights the symmetric retro-1,2-addition part. The third animation emphasizes a 1,3-C atom shift of the hypovalent C atom across the ethenyl group. Otherwise, the IRCs leading to carbene **9** are indistinguishable.

**Figure 3:**
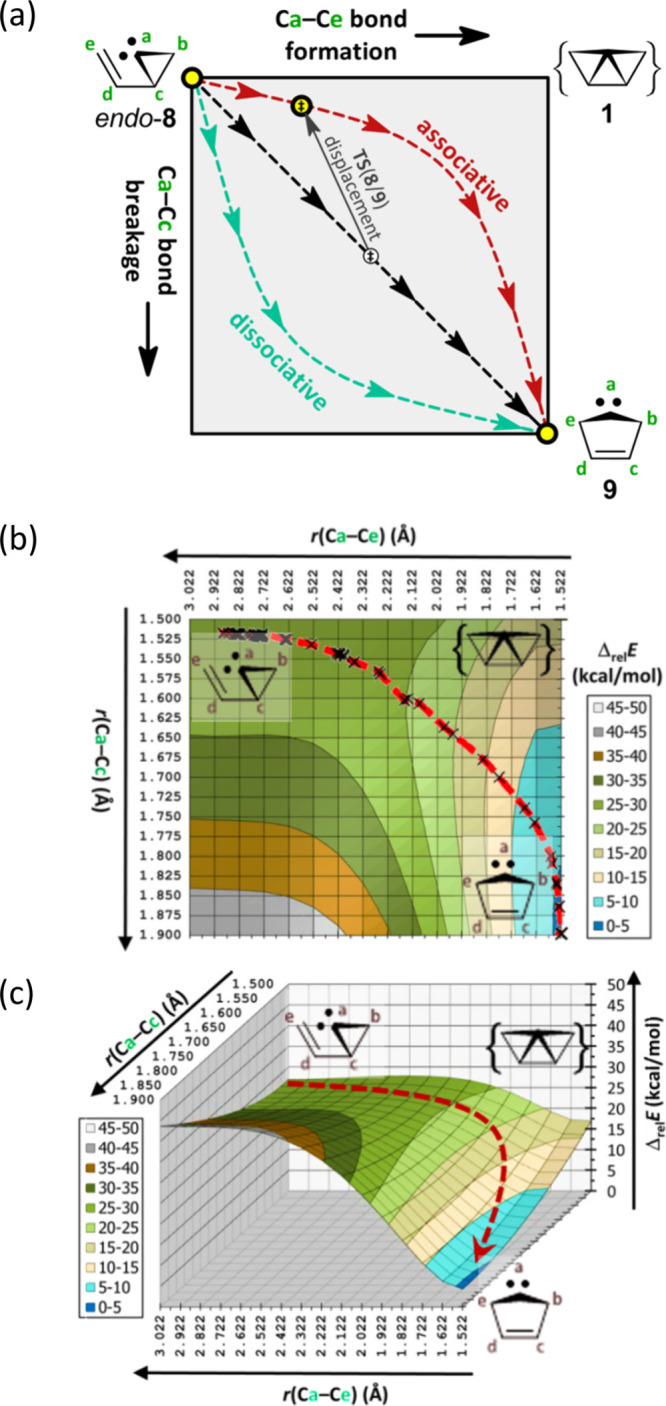
Elementary step *endo*-**8** → **9** is outlined in (a) a More O’Ferrall–Jencks diagram computed to give (b) a 2D contour map and (c) the corresponding 3D surface showing the IRC pathway (dashed red curve) [ωB97X-D/def2-TZVP].

## Conclusions

The divalent C atom of carbene *endo*-**8** is ready to form **1** via a rapid intramolecular 1,2-addition because the neighboring ethenyl group participates in *two* donor → acceptor FMO interactions, a π-complex (π → p) *and* a reinforcing σ-complex (Walsh → π^*^), that anchimerically stabilize **TS**(**8**/**9**) reducing the *E*_a_ to just 1.2 kcal/mol. Cyclopropanation is frustrated past **TS**(**8**/**9**), however, because **1** may be a TS itself or represent a nonequilibrium geometry. Generating **1** from nonclassical carbene **9** is also foiled. Ultimately, symmetric retro-1,2-addition ensues—*as if* **1** *had been attained*—leading to **9** in a single but asynchronous elementary step. The minimum-energy path on the PES representing ring-expansion *endo*-**8** → **9** approaches **1** and most closely resembles the predicted associative curve of the corresponding More O’Ferrall–Jencks diagram. During the Skattebo̷l rearrangement of *endo*-**8**, *C*_*s*_-symmetric **9** flattens to the lower-energy *C*_2*v*_-symmetric carbene **6**. Cyclic diene **7** is ultimately produced by a 1,2-H atom shift within carbene **6**.

## Experimental Section

### Computational Methods

Computations were performed using the *Spartan’24* (v. 1.2.0) software package.^29^ Equilibrium geometries, transition states, intrinsic reaction coordinates, step calculations, and 2D grid plots were computed using the RSH-GGA (U)­ωB97X-D DFT functional with a triple-ζ def2-TZVP Ahlrichs–Weigend basis set with an integration size of 100 radial points and 434 Lebedev grid points.^18,19^ 2,3-Secopyramidane was also modeled using the DLFF3 molecular mechanics force-field.^30^ Normal-mode vibrational analyses were performed at the level of geometry optimization, except for 2,3-secopyramidane (DLFF3). The harmonic frequencies were used to obtain temperature-independent zero-point vibrational energy (*E*_ZPV_)^31^ and temperature-dependent (*T* = 298.15 K) thermal vibrational energy (Δ_vib_ *H*) values. Each transition state had one, and only one, imaginary frequency ( ${\mathrm{\nu ̅}}_{\mathrm{TS}}$ ) whose normal mode was animated to verify the motions expected for the elementary step, which was confirmed by an intrinsic reaction coordinate calculation. All *E*_ZPV_ values were scaled by z = 0.955 ^32^ before being added to *E* (*T* = 0 K; *p* = 0 atm). Relative energy values (Δ_rel_ *E*) are specified with respect to planar cyclopent-3-en-1-ylidene, which was set equal to [0] kcal/mol. The increase in kinetic energy, due to translations (3­(1/2)­R*T*) and rotations (3­(1/2)­R*T*), for each nonlinear molecule was then added. Finally, R*T* (i.e., “*pV* work” needed to expand one mole of ideal gas to *V* = 24.465 L at *T* = 298.15 K and *p* = 1 atm) was added to obtain *H*_*T*_ (see Supporting Information Table S1, eq S1).

## Supplementary Material











## Data Availability

The data underlying this study are available in the published article and its online Supporting Information.

## Notes

Schemes S1–S7, Figures S1–S8, Tables S1–S5, energy profiles via IRC and step calculations, grid calculations with 2D contour and 3D surface plots, Cartesian coordinates and molecular energies (PDF)
Energy values (XLSX)
Graph data (XLSX)
Energy surfaces (XLSX)
Videos S1–S11 (ZIP)

## ∇

*Dedikert til Professor Lars Skattebo̷l, Universitetet i Oslo, for hans banebrytende arbeid innen karbenkjemi*. (Nor.) (Dedicated to Professor Lars Skattebo̷l, University of Oslo, for his pioneering work in carbene chemistry.)
