# Supplementary material for: Quest for 2,3-Secopyramidane: Computations Hint at Elusive Structure during Skattebo̷l Rearrangement of Vinylcyclopropylidene
Source: J Org Chem. 2026 Apr 15;91(16):5699–704. doi: 10.1021/acs.joc.5c03248 (PMC13122649; doi:10.1021/acs.joc.5c03248)
Supplement: Supplementary file 1 [file jo5c03248_si_001.pdf]

Quest for 2,3-Secopyramidane: Computations Hint at Elusive Structure during Skattebøl Rearrangement of Vinylcyclopropylidene§,∇

Murray G. Rosenberg† and Udo H. Brinker*,‡

† *Independent Researcher, Johnson City, NY 13790, United States*

‡ *Institute of Organic Chemistry, University of Vienna, Vienna 1090, Austria*

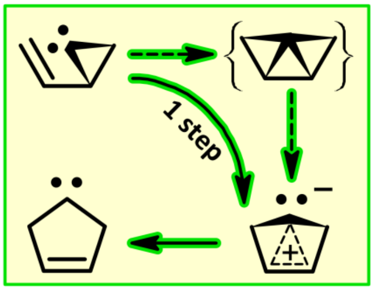

Table of Contents

Page

Physical Constants S2

Secopyramidanes S2

Frontier MOs of *endo*-**8**, **TS**(**8**/**9**), and **9** S3

Intrinsic Reaction Coordinate (IRC) Diagrams S4

Allene Formation S8

Ethenyl Group Rotation S9

Cartesian Coordinates and Molecular Energies S11

ORTEP Structures S23

Relative Thermodynamic Values S24

§ CARBENE REARRANGEMENTS. XCVI. For PART XCV, see: Rosenberg, M. G.; Brinker, U. H. Carbene Routes to Cyclopropatetrahedrane. *J. Org. Chem.* **2022,** *87,* 16902−16906.

∇ *Dedikert til Professor Lars Skattebøl, Universitetet i Oslo, for hans banebrytende arbeid innen karbenkjemi. (Nor.)* (Dedicated to Professor Lars Skattebøl, University of Oslo, for his pioneering work in carbene chemistry.)

Physical Constants

Table S1. Values Used in Computations with Equation S1

Parameter		Value
V	=	nRT/p
	=	24.465 L
p	=	1 atm
n	=	1 mol
R	=	1.9872×10^{-3} (kcal/mol)/K
	=	8.2057×10^{-2} (L·atm/mol)/K
T	=	298.15 K
	=	25 °C
RT	=	0.592 kcal/mol
3(½)RT	=	0.889 kcal/mol
1 hartree	=	627.5095 kcal/mol

$$H_T = [E + \text{Z}(E_{\text{ZPV}})] + \text{H}(H_{\text{vib}}) + (3(½)\text{R}T)_{\text{translational}} + (3(½)\text{R}T)_{\text{rotational}} + (\text{R}T)_{\text{ideal gas}} \quad \text{(S1)}$$

Scheme S1. Conceptual Formation of Secopyramidanes from Pyramidane

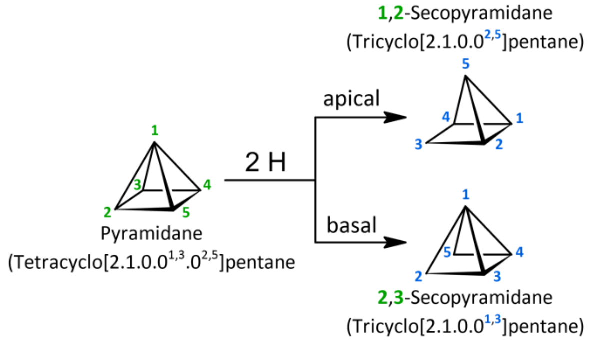

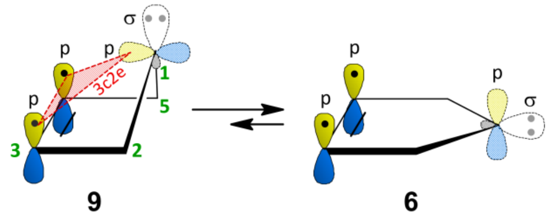

Figure S1. Equilibrium between nonclassical and classical cyclopent-3-en-1-ylidenes **9** and **6**, respectively. Ring-puckered **9** features delocalized 3c2e bonding making it bis-homoaromatic. Planar **6** is a classical carbene with traditional bonding.

Table S2. Frontier MOs of *endo*-2-Ethenylcycloprop-1-ylidene (*endo*-8), TS(8/9), and Nonclassical Cyclopent-3-en-1-ylidene (9)

Orbital	*endo*-**8** (reverse)[a]	*endo*-**8** (obverse)[a]	*endo*-**8**	**TS**(**8**/**9**)	**9**
**LUMO{+1}**	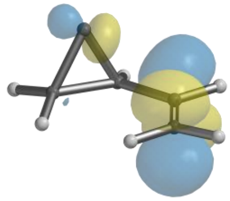	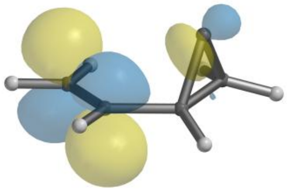	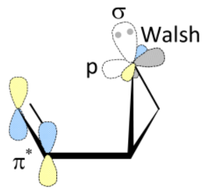<br>1.9 eV	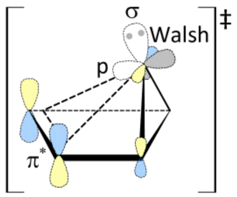<br>2.1 eV	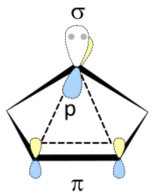<br>2.1 eV
**LUMO**	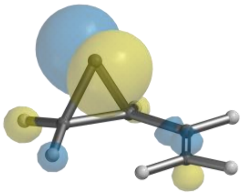	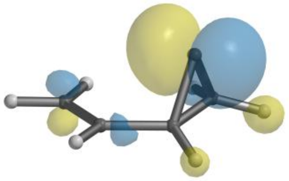	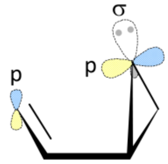<br>−0.6 eV	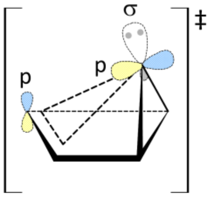<br>0.1 eV	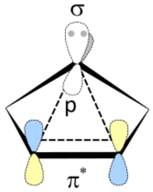<br>1.5 eV
**HOMO**	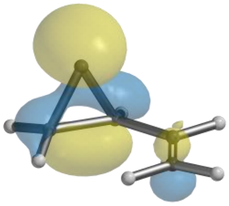	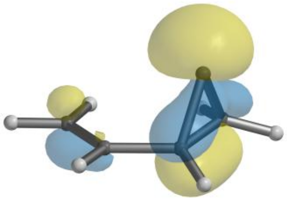	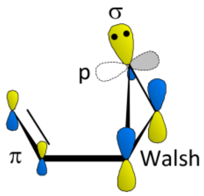<br>−8.6 eV	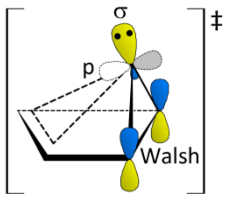<br>−8.7 eV	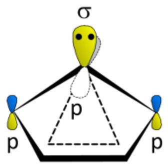<br>−7.8 eV
**HOMO{−1}**	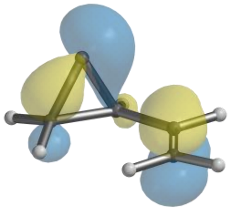	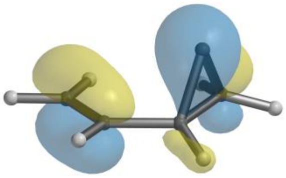	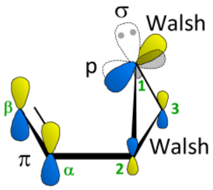<br>−9.5 eV	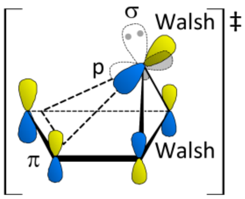<br>−8.7 eV	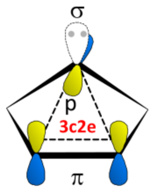<br>−10.2 eV

[a] MO surfaces and energies computed using the ωB97X-D/def2-TZVP theoretical model.

Isosurface value = $0.064\sqrt{\frac{e}{a_0^3}}$

Intrinsic Reaction Coordinate Diagrams

Step-by-step IRCs following the Skattebøl rearrangement.

Scheme S2. First Step of *endo*-2-Ethenylcycloprop-1-ylidene (*endo*-8) Skattebøl Rearrangement

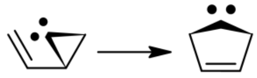

endo-**8** **9**

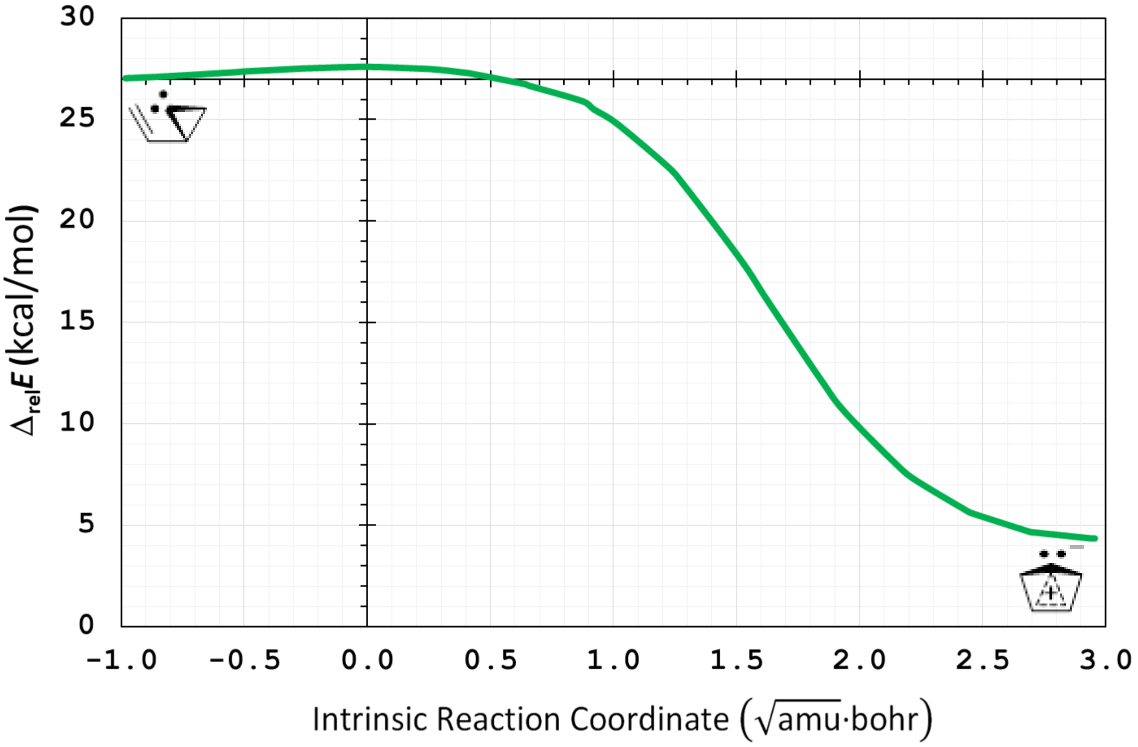

Figure S2. IRC for *endo*-**8**→**9**. An input of E_a = 1.2 kcal/mol is required for the ring-expansion reaction. [ωB97X-D/def2-TZVP]

Scheme S3. Equilibrium between Carbenes 9 and 6

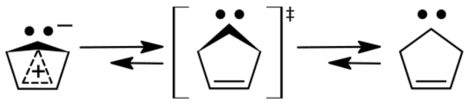

9 TS(9/6) 6

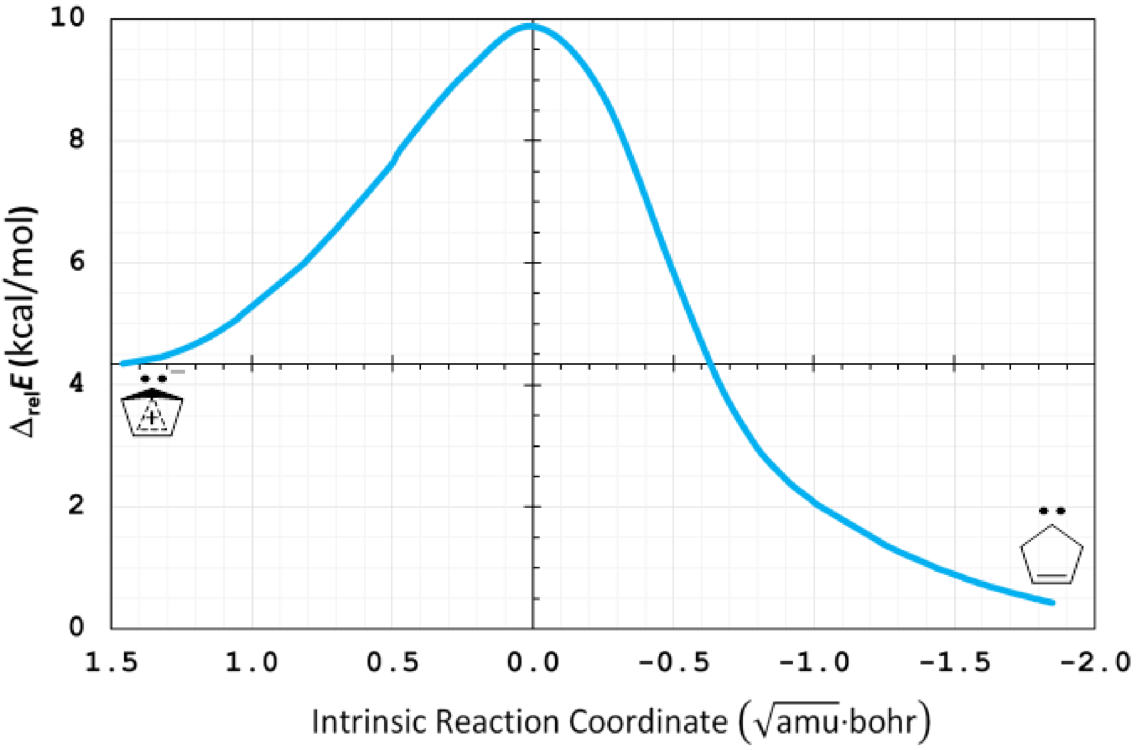

Figure S3. IRC for **9**→**6**. An input of E_a = 4.6 kcal/mol is required to flatten bis-homoaromatic carbene **9** to carbene **6**. [ωB97X-D/def2-TZVP]

Scheme S4. Retro-1,2-Addition of 2,3-Secopyramidane (1)[a]

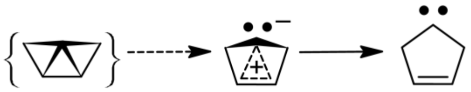

1 9 6

[a] 2,3-Secopyramidane via molecular mechanics [DLFF3].

(a)

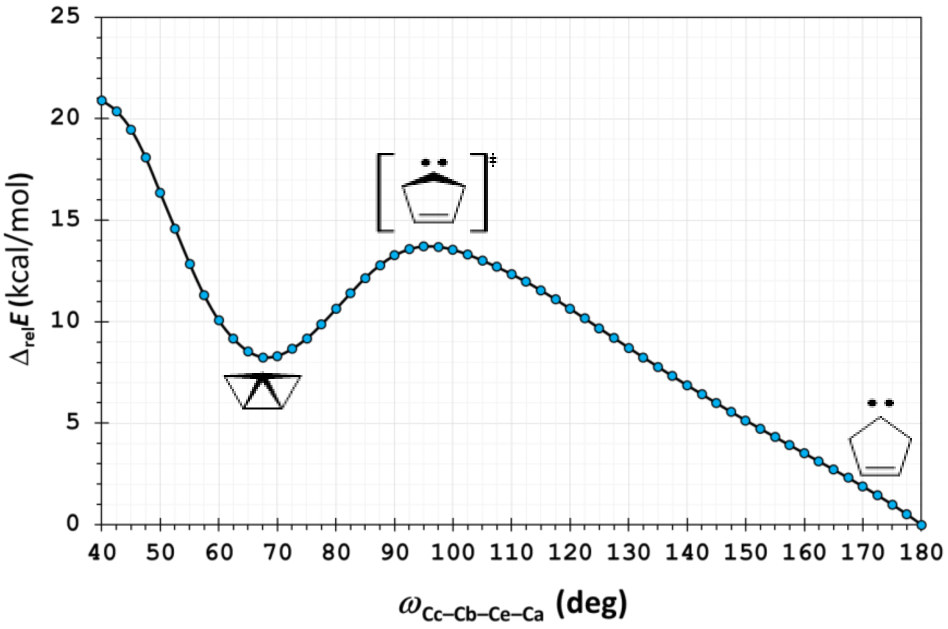

(b)

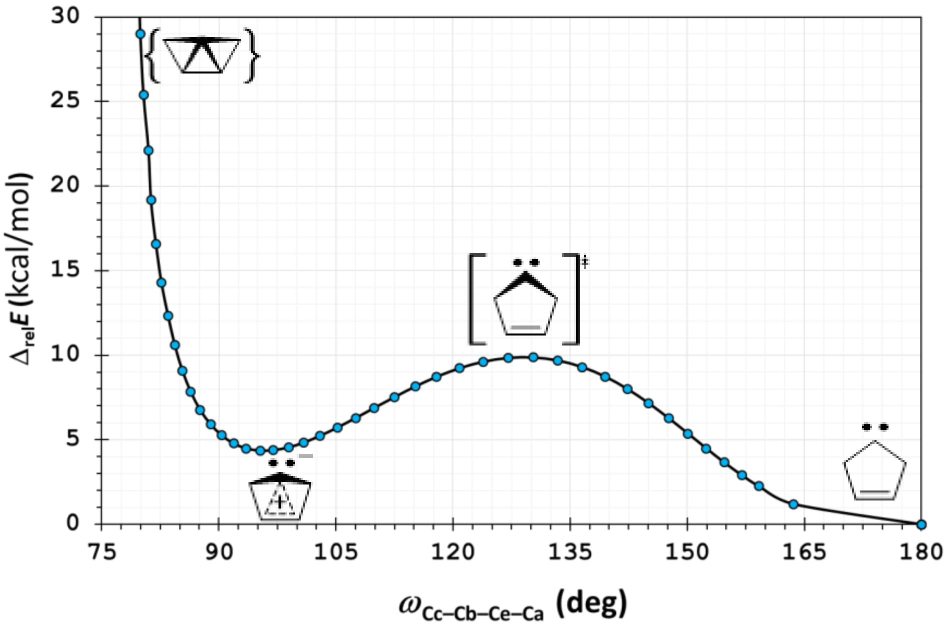

Figure S4. Semi-rigid energy profiles for the flattening of 2,3-secopyramidane (**1**) using (a) molecular mechanics [DLFF3] versus (b) density functional theory [ωB97X-D/def2-TZVP].

Scheme S5. Carbene 6 Undergoes a 1,2-H Atom Shift to Cyclic Diene 7

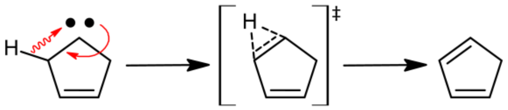

6 **TS(6/7)** **7**

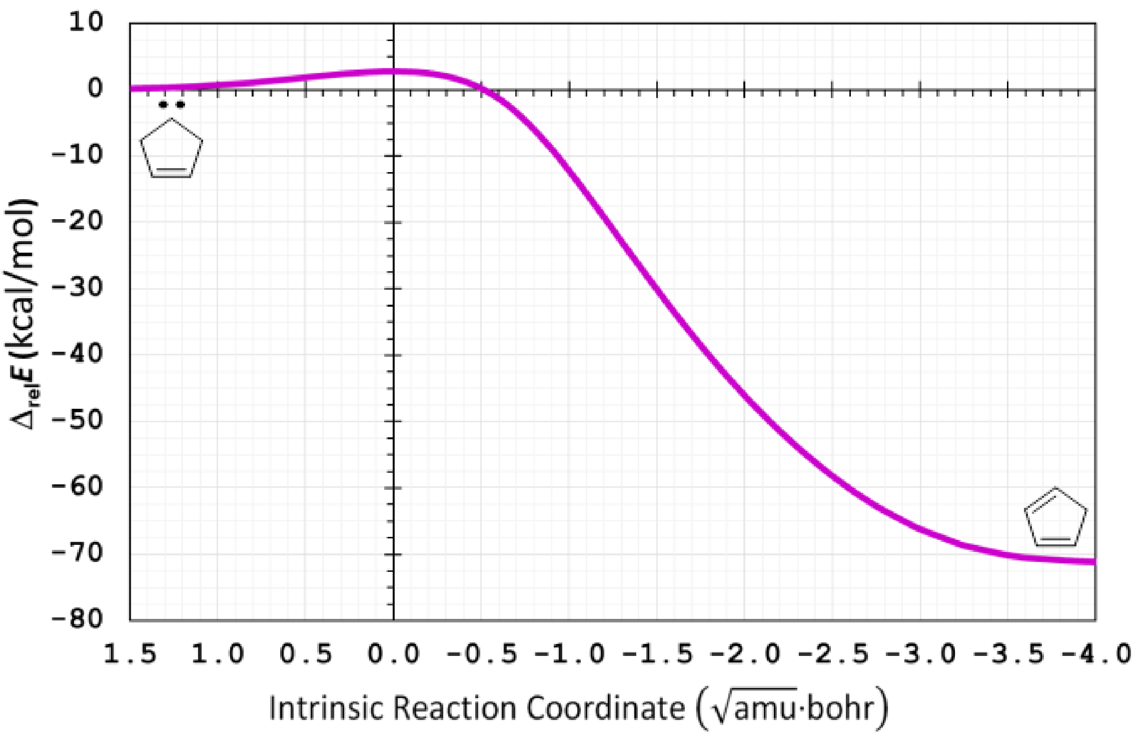

Figure S5. IRC for **6**→**7**. An input of E_a = 2.1 kcal/mol is required to initiate 1,2-H atom shift. [ωB97X-D/def2-TZVP]

Allene Formation

The Skattebøl rearrangement occurs as low as T = –78 °C but the amount of penta-1,2,4-triene (**10**) increases as T increases (Scheme S6; Figure S6).

Scheme S6. Ring-Opening of 2-Ethenylcycloprop-1-ylidene (8) to Penta-1,2,4-triene (10)

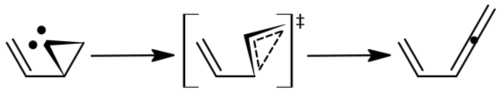

endo-**8** **TS**(**8**/**10**) *s-cis*-**10**

(a)

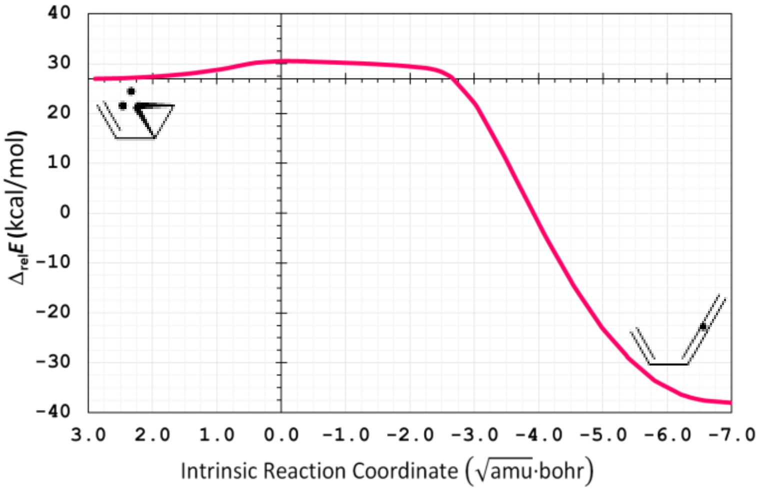

(b)

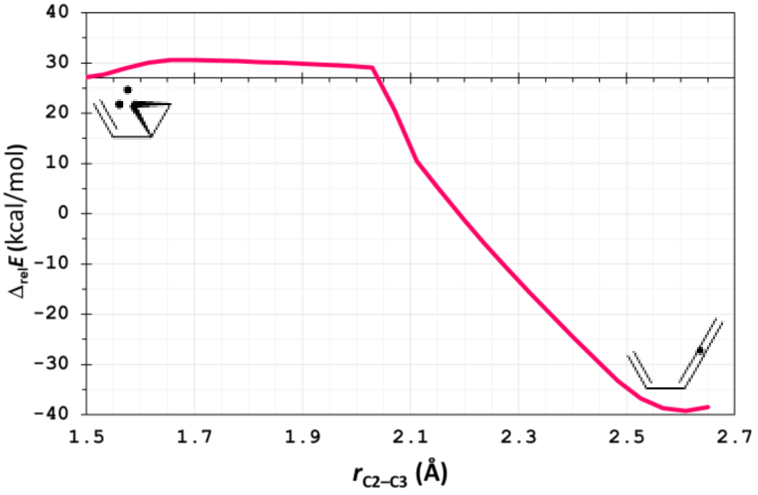

Figure S6. Formation of allene *s-cis*-**10** from carbene *endo*-**8**: (a) IRC for *endo*-**8**→*s-cis*-**10**. An input of E_a = 3.3 kcal/mol is required to open cyclic carbene *endo*-**8**. (b) Step-calculation produced by increasing distance r(Cb–Cc) within *endo*-**8**. [ωB97X-D/def2-TZVP]

Ethenyl Group Rotation

Rotamerization within 2-ethenylcycloprop-1-ylidene (**8**) is outlined in Scheme S7. Step calculations using dihedral angle increments ($\Delta\omega$) of 5 deg were computed using the ωB97X-D/def2-TZVP theoretical model and plotted using Cartesian coordinates and polar coordinates (Figure S7).

Scheme S7. Pendant Group Rotation within 2-Ethenylcycloprop-1-ylidene (8)

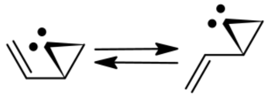

endo-**8** *exo*-**8**

(a) (b)

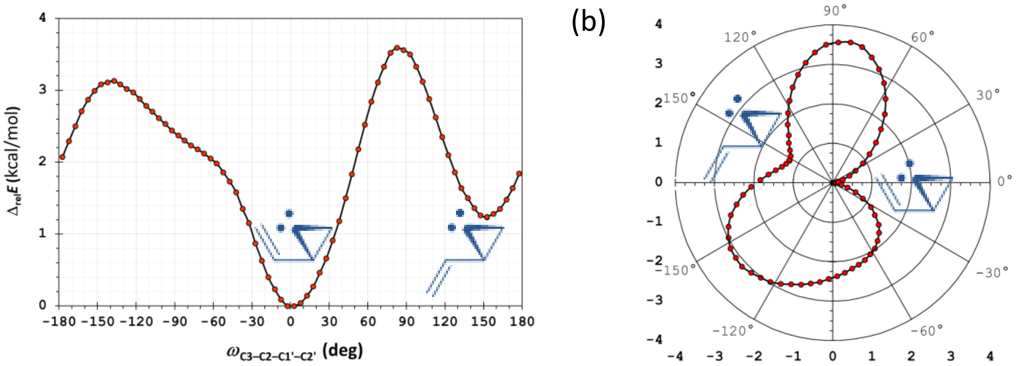

Figure S7. Ethenyl group rotation interconverts *endo*-**8** to *exo*-**8**. Each point represents a dihedral angle increment ($\Delta\omega$) of 5 deg, shown using (a) Cartesian coordinates and (b) polar coordinates. [ωB97X-D/def2-TZVP]

Table S3. Grid Plot Energy Values[a,b]

ΔE (kcal/mol)		r (Ca-(	r (Ca-(	r (Ca-(	r (Ca-(	r (Ca-(	r (Ca-(	r (Ca-(	r (Ca-(	r (Ca-(	r (Ca-(	r (Ca-(	r (Ca-(	r (Ca-(	r (Ca-(	r (Ca-(	r (Ca-(
		3.022	2.922	2.822	2.722	2.622	2.522	2.422	2.322	2.222	2.122	2.022	1.922	1.822	1.722	1.622	1.522
r (Ca-Cc) (Å)	1.500	26.962	27.026	27.193	27.422	27.652	27.801	27.825	27.727	27.407	26.742	25.571	23.824	21.583	19.178	17.218	16.938
r (Ca-Cc) (Å)	1.525	27.007	27.024	27.163	27.381	27.536	27.602	27.526	27.258	26.739	25.854	24.459	22.491	20.085	17.558	15.565	15.359
r (Ca-Cc) (Å)	1.550	27.247	27.255	27.381	27.547	27.659	27.669	27.468	27.043	26.328	25.221	23.599	21.408	18.813	16.158	14.095	13.933
r (Ca-Cc) (Å)	1.575	27.726	27.716	27.797	27.925	28.004	27.928	27.624	27.040	26.133	24.807	22.957	20.541	17.751	14.924	12.780	12.632
r (Ca-Cc) (Å)	1.600	28.374	28.344	28.412	28.511	28.563	28.381	27.960	27.226	26.129	24.586	22.505	19.866	16.865	13.869	11.606	11.434
r (Ca-Cc) (Å)	1.625	29.187	29.149	29.184	29.255	29.225	28.994	28.454	27.579	26.293	24.537	22.221	19.359	16.142	12.958	10.555	10.322
r (Ca-Cc) (Å)	1.650	30.125	30.069	30.092	30.126	30.042	29.744	29.093	28.071	26.612	24.636	22.090	19.002	15.568	12.183	9.626	9.303
r (Ca-Cc) (Å)	1.675	31.181	31.115	31.123	31.106	30.982	30.605	29.859	28.692	27.049	24.874	22.105	18.788	15.132	11.537	8.803	8.370
r (Ca-Cc) (Å)	1.700	32.331	32.257	32.240	32.204	32.032	31.580	30.738	29.424	27.614	25.234	22.241	18.697	14.814	11.010	8.101	7.532
r (Ca-Cc) (Å)	1.725	33.564	33.483	33.450	33.389	33.169	32.651	31.707	30.267	28.293	25.710	22.501	18.732	14.617	10.592	7.498	6.786
r (Ca-Cc) (Å)	1.750	34.862	34.776	34.732	34.647	34.387	33.803	32.769	31.205	29.061	26.287	22.864	18.877	14.536	10.291	7.009	6.131
r (Ca-Cc) (Å)	1.775	36.237	36.137	36.083	35.972	35.681	35.040	33.916	32.233	29.934	26.978	23.340	19.132	14.560	10.105	6.618	5.583
r (Ca-Cc) (Å)	1.800	37.633	37.541	37.484	37.363	37.037	36.344	35.141	33.341	30.891	27.745	23.914	19.493	14.693	10.034	6.346	5.146
r (Ca-Cc) (Å)	1.825	39.089	39.001	38.945	38.808	38.459	37.720	36.441	34.541	31.936	28.615	24.584	19.937	14.917	10.024	6.168	4.807
r (Ca-Cc) (Å)	1.850	40.569	40.499	40.445	40.307	39.933	39.152	37.797	35.791	33.057	29.564	25.337	20.474	15.230	10.133	6.090	4.552
r (Ca-Cc) (Å)	1.875	42.096	42.034	41.986	41.869	41.454	40.635	39.223	37.119	34.248	30.590	26.171	21.091	15.627	10.328	6.121	4.408
r (Ca-Cc) (Å)	1.900	43.639	43.600	43.566	43.432	43.032	42.176	40.709	38.506	35.512	31.685	27.074	21.782	16.104	10.614	6.231	4.352

[a] ωB97X-D/def2-TZVP theoretical model. [b] $\Delta_{rel}E$(**6**) = [0] kcal/mol.

Cartesian Coordinates and Molecular Energies

2,3-Secopyramidane (**1**); *cis*-Tricyclo[2.1.0.0^{1,3}]pentane; ($\bar{\nu}_{TS}$ = 335*i* cm^{-1})
ωB97X-D/def2-TZVP//DLFF3 + 0.955(E_{ZPV}):

<u>*T* = 0 K</u>

E = −193.936530 hartree
E_{ZPV} = 57.6151 kcal/mol
E + z(E_{ZPV}) = −121641.99 kcal/mol

<u>*T* = 298.15 K</u>

H_T = −121638.68 kcal/mol

Atom	*x*	*y*	*z*
C	-0.815285	-0.768526	0.000000
C	0.015621	0.801019	0.678932
C	0.015621	0.801019	-0.678932
C	0.128919	-0.593724	-1.181344
C	0.128919	-0.593724	1.181344
H	-0.332432	1.636778	1.271807
H	-0.332432	1.636778	-1.271807
H	-0.303053	-0.736448	-2.166913
H	-0.303053	-0.736448	2.166913
H	1.120453	-1.048318	-1.095644
H	1.120453	-1.048318	1.095644

Nonclassical Cyclopent-3-en-1-ylidene (**9**); (C_s)
ωB97X-D/def2-TZVP + 0.955(E_{ZPV}):

<u>T = 0 K</u>

E = −193.983907 hartree
E_{ZPV} = 57.7035 kcal/mol
E + z(E_{ZPV}) = −121671.63 kcal/mol

<u>T = 298.15 K</u>

H_T = −121668.30 kcal/mol

Atom	*x*	*y*	*z*
C	-0.760848	-0.694254	0.000000
C	0.084040	0.867472	0.678947
C	0.084040	0.867472	-0.678947
C	0.185099	-0.528222	-1.181268
C	0.185099	-0.528222	1.181268
H	-0.256776	1.706176	1.271867
H	-0.256776	1.706176	-1.271867
H	-0.247995	-0.667180	-2.166878
H	-0.247995	-0.667180	2.166878
H	1.172486	-0.991730	-1.095422
H	1.172486	-0.991730	1.095422

exo-2-Ethenylcycloprop-1-ylidene (*exo*-**8**);
ωB97X-D/def2-TZVP + 0.955(E_{ZPV}):

<u>*T* = 0 K</u>

E = −193.945907 hartree
E_{ZPV} = 54.9041 kcal/mol
E + z(E_{ZPV}) = −121650.46 kcal/mol

<u>*T* = 298.15 K</u>

H_T = −121646.54 kcal/mol

Atom	*x*	*y*	*z*
C	-1.619754	0.873251	0.567532
C	-1.349843	-0.481070	1.096690
C	-0.621567	-0.023529	-0.117990
C	0.808323	0.340364	-0.098113
C	1.622703	0.254039	-1.139873
H	-2.139849	-1.220145	1.017375
H	-0.991301	-0.387183	-1.074165
H	-0.844731	-0.489930	2.060154
H	1.193257	0.699571	0.852029
H	1.278770	-0.101475	-2.104309
H	2.663991	0.536104	-1.059331

Triplet *endo*-2-Ethenylcycloprop-1-ylidene ([3]**8**); Triplet Bicyclo[2.1.0]pentane-1,3-diyl, <S^2> = 2.0098
ωB97X-D/def2-TZVP + 0.955(E_{ZPV}):

<u>*T* = 0 K</u>

E = −193.932796 hartree
E_{ZPV} = 56.2299 kcal/mol
E + z(E_{ZPV}) = −121640.97 kcal/mol

<u>*T* = 298.15 K</u>

H_T = −121637.57 kcal/mol

Atom	*x*	*y*	*z*
C	-1.108313	-0.825295	0.099216
C	-0.609270	0.408993	-0.707891
C	-0.532495	0.329752	0.791855
C	0.867727	0.426945	-0.722933
C	0.989012	0.407241	0.789007
H	-2.189572	-0.902283	0.120507
H	-1.293616	0.914389	-1.375938
H	-0.580799	-1.772504	0.034304
H	1.365949	1.352713	1.187657
H	1.527150	-0.418919	1.262255
H	1.564226	0.078969	-1.478039

endo-2-Ethenylcycloprop-1-ylidene (*endo*-**8**);
ωB97X-D/def2-TZVP + 0.955(E_{ZPV}):

<u>*T* = 0 K</u>

E = −193.947902 hartree
E_{ZPV} = 55.2331 kcal/mol
E + z(E_{ZPV}) = −121651.40 kcal/mol

<u>*T* = 298.15 K</u>

H_T = −121647.56 kcal/mol

Atom	*x*	*y*	*z*
C	-1.389480	0.871030	-0.181127
C	-1.344806	-0.417032	0.546041
C	-0.559654	-0.278503	-0.703968
C	0.849642	0.162399	-0.665111
C	1.534492	0.447864	0.437294
H	-2.269374	-0.979736	0.445736
H	-0.916559	-0.464969	1.541949
H	-0.884567	-0.801830	-1.593819
H	1.122321	0.317998	1.429953
H	1.307125	0.329249	-1.634361
H	2.550859	0.813529	0.377413

TS_*endo*-2-Ethenylcycloprop-1-ylidene/Nonclassical Cyclopent-3-en-1-ylidene (**TS**(**8**/**9**)); ($\bar{\nu}_{TS}$ = 151*i* cm^{-1})
ωB97X-D/def2-TZVP + 0.955(E_{ZPV}):

T = 0 K

E = −193.946853 hartree
E_{ZPV} = 55.7755 kcal/mol
E + z(E_{ZPV}) = −121650.22 kcal/mol

T = 298.15 K

H_T = −121646.49 kcal/mol

Atom	*x*	*y*	*z*
C	-1.246331	-0.415056	0.553596
C	-1.022921	0.900104	-0.135601
C	-0.559169	-0.423776	-0.747148
C	0.814512	0.064819	-0.676957
C	1.401833	0.483623	0.456608
H	-2.304892	-0.658330	0.507167
H	-0.936067	-0.845281	-1.666063
H	-0.770909	-0.711318	1.482897
H	0.996204	0.298300	1.439084
H	1.279671	0.300176	-1.627684
H	2.348071	1.006739	0.414100

Cyclopent-3-en-1-ylidene (**6**); (C_{2v})
ωB97X-D/def2-TZVP + 0.955(E_{ZPV}):

<u>T = 0 K</u>

E = −193.990842 hartree
E_{ZPV} = 55.8694 kcal/mol
E + z(E_{ZPV}) = −121677.73 kcal/mol

<u>T = 298.15 K</u>

H_T = −121674.23 kcal/mol

Atom	*x*	*y*	*z*
C	-1.169950	0.000000	-0.417677
C	-0.662992	0.000000	1.002755
C	0.000000	0.000000	-1.337590
C	0.662992	0.000000	1.002755
C	1.169950	0.000000	-0.417677
H	-1.802824	-0.859826	-0.679387
H	-1.802824	0.859826	-0.679387
H	-1.297178	0.000000	1.879378
H	1.297178	0.000000	1.879378
H	1.802824	-0.859826	-0.679387
H	1.802824	0.859826	-0.679387

TS_Cyclopent-3-en-1-ylidene/Cyclopenta-1,3-diene (**TS**(**6**/**7**)); ($\bar{\nu}_{TS}$ = 551*i* cm^{-1})
ωB97X-D/def2-TZVP + 0.955(E_{ZPV}):

T = 0 K

E = −193.986379 hartree
E_{ZPV} = 55.1747 kcal/mol
E + z(E_{ZPV}) = −121675.60 kcal/mol

T = 298.15 K

H_T = −121672.08 kcal/mol

Atom	*x*	*y*	*z*
C	-1.163864	0.231617	-0.429165
C	-0.662249	0.143938	0.974223
C	-0.105011	0.193027	-1.387730
C	0.654547	-0.037263	0.923600
C	1.098427	-0.070674	-0.512696
H	-2.159841	0.588057	-0.676080
H	-1.278721	0.206434	1.859699
H	-1.126847	-0.822528	-0.978926
H	1.314180	-0.148174	1.773612
H	1.601228	-0.999848	-0.800887
H	1.828150	0.715413	-0.745650

TS_Nonclassical Cyclopent-3-en-1-ylidene/Cyclopent-3-en-1-ylidene (**TS**(**9**/**6**)); ($\bar{\nu}_{TS}$ = 414*i* cm^{-1})
ωB97X-D/def2-TZVP + 0.955(E_{ZPV}):

<u>*T* = 0 K</u>

E = −193.975097 hartree
E_{ZPV} = 56.7637 kcal/mol
E + z(E_{ZPV}) = −121667.00 kcal/mol

<u>*T* = 298.15 K</u>

H_T = −121663.60 kcal/mol

Atom	*x*	*y*	*z*
C	-0.533604	-1.155818	0.000000
C	0.005086	0.911352	0.665636
C	0.005086	0.911352	-0.665636
C	0.131440	-0.508309	-1.179785
C	0.131440	-0.508309	1.179785
H	-0.367306	-0.694847	-2.129358
H	-0.367306	-0.694847	2.129358
H	-0.106682	1.779172	1.301602
H	-0.106682	1.779172	-1.301602
H	1.187731	-0.809294	-1.273128
H	1.187731	-0.809294	1.273128

Cyclopenta-1,3-diene (**7**);
ωB97X-D/def2-TZVP + 0.955(E_{ZPV}):

<u>T = 0 K</u>

E = −194.104252 hartree
E_{ZPV} = 58.4477 kcal/mol
E + z(E_{ZPV}) = −121746.44 kcal/mol

<u>T = 298.15 K</u>

H_T = −121743.26 kcal/mol

Atom	*x*	*y*	*z*
C	-1.172258	0.000000	-0.280353
C	-0.732090	0.000000	0.984206
C	0.000000	0.000000	-1.210988
C	0.732090	0.000000	0.984206
C	1.172258	0.000000	-0.280353
H	-2.202028	0.000000	-0.605799
H	-1.346755	0.000000	1.873472
H	0.000000	-0.876694	-1.868400
H	0.000000	0.876694	-1.868400
H	1.346755	0.000000	1.873472
H	2.202028	0.000000	-0.605799

TS_*endo*-2-Ethenylcycloprop-1-ylidene/*s*-*cis*-Penta-1,2,4-triene (**TS**(**8**/**10**)); ($\bar{\nu}_{TS}$ = 173*i* cm^{-1})
ωB97X-D/def2-TZVP + 0.955(E_{ZPV}):

<u>*T* = 0 K</u>

E = −193.942212 hartree
E_{ZPV} = 54.9072 kcal/mol
E + z(E_{ZPV}) = −121648.14 kcal/mol

<u>*T* = 298.15 K</u>

H_T = −121644.18 kcal/mol

Atom	*x*	*y*	*z*
C	-1.757074	-0.027307	0.203907
C	-0.980776	1.078978	0.622532
C	-0.339584	0.526955	-0.537883
C	1.065573	0.100914	-0.460576
C	1.637919	-0.388285	0.632768
H	-2.357985	-0.141205	-0.690714
H	-1.940483	-0.792772	0.957014
H	-0.731538	0.625254	-1.543488
H	1.074391	-0.530870	1.546993
H	1.644383	0.209029	-1.372528
H	2.685174	-0.660691	0.641973

s-cis-Penta-1,2,4-triene (*s-cis*-**10**);
ωB97X-D/def2-TZVP + 0.955(E_{ZPV}):

<u>*T* = 0 K</u>

E = −194.053443 hartree
E_{ZPV} = 56.2580 kcal/mol
E + z(E_{ZPV}) = −121716.65 kcal/mol

<u>*T* = 298.15 K</u>

H_T = −121712.75 kcal/mol

Atom	*x*	*y*	*z*
C	-2.156701	0.461173	0.000000
C	-1.068722	-0.245221	0.000000
C	0.033950	-0.944669	0.000000
C	1.394963	-0.387817	0.000000
C	1.712075	0.901575	0.000000
H	-2.628199	0.765405	0.928083
H	-2.628199	0.765405	-0.928083
H	-0.057217	-2.026358	0.000000
H	0.952143	1.674872	0.000000
H	2.190689	-1.125081	0.000000
H	2.745895	1.219835	0.000000

Table S4. Structural Changes during Skattebøl Rearrangement of Carbene *endo*-8

Dimension	*endo*-**8** [a]	**TS(8/9)** [a]	**9** [a]	**6** [a]	**1** [b]
r(Cb−Cc) (Å)	1.483	1.471	1.487	1.508	1.461
r(Ca−Cb) (Å)	1.480	1.502	1.522	1.488	1.489
r(Ca−Cc) (Å)	1.511	1.530	1.901	2.432	1.534
r(Ca−Cd) (Å)	2.398	2.090	1.901	2.432	1.534
r(Ca−Ce) (Å)	3.018	2.531	1.522	1.488	1.489
r(Cd−Ce) (Å)	1.329	1.344	1.487	1.508	1.461
r(Cc−Cd) (Å)	1.477	1.460	1.358	1.326	1.449
θ(Cc−Ca−Cd) (deg)	36.15	44.29	41.85	31.63	56.34
θ(Cb−Ca−Cc) (deg)	59.42	58.05	49.99	36.01	57.74
θ(Ce−Ca−Cd) (deg)	25.23	32.04	49.99	36.01	57.74
ω(Cc−Cb−Ce−Ca) (deg)	64.53	70.56	95.81	180.00	70.24

[a] Computed using the ωB97X-D/def2-TZVP theoretical model. [b] Computed using molecular mechanics [DLFF3].

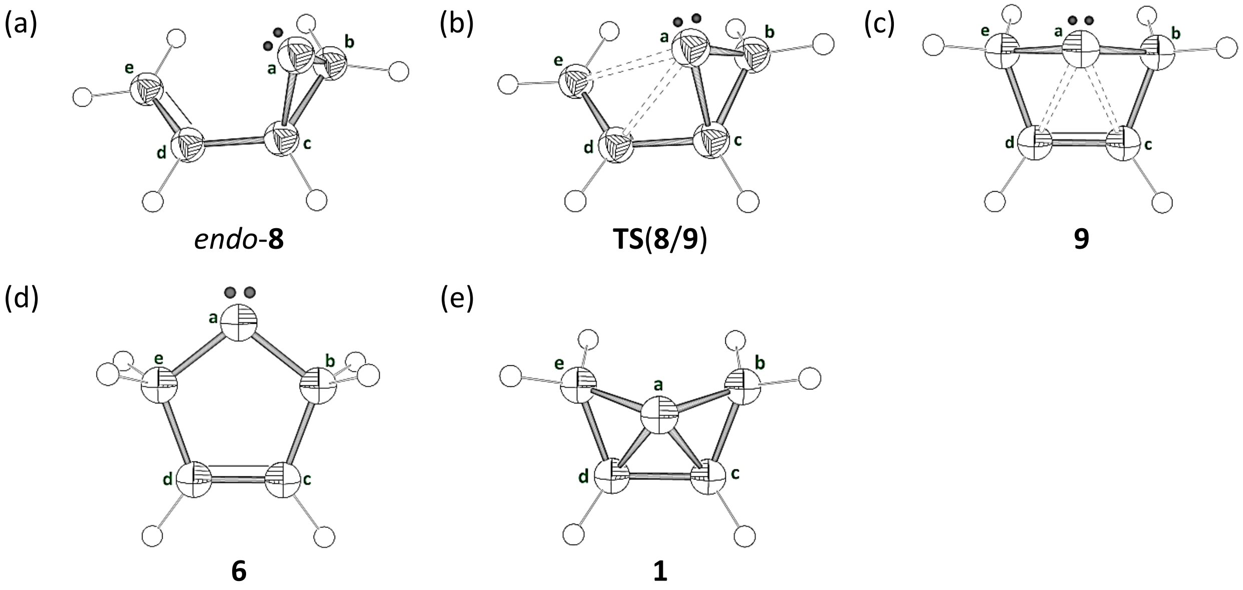

Figure S8. ORTEP figures (50% ellipsoids) of (a) *endo*-**8**, (b) **TS(8/9)**, (c) **9**, (d) **6**, and (e) **1**.

Table S5. Relative Thermodynamic Values[a]

Compound	$\Delta E + 0.955(E_{ZPV})$ (kcal/mol)	ΔH^b (kcal/mol)	ΔS^b (cal/mol)/K	ΔG^b (kcal/mol)
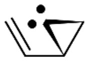 *endo*-2-ethenylcycloprop-1-ylidene (*endo*-**8**)	26.34	26.66	71.37	25.59
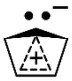 nonclassical cyclopent-3-en-1-ylidene (**9**)	6.10	5.92	67.25	6.07
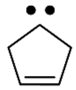 cyclopent-3-en-1-ylidene (**6**)	[0]	[0]	67.75	[0]
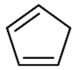 cyclopenta-1,3-diene (**7**)	−68.70	−69.03	65.23	−68.28
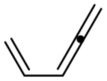 *s-cis*-penta-1,2-4-triene (*s-cis*-**10**)	−38.91	−38.52	72.28	−39.87
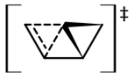 **TS**(**8**/**9**) $\bar{\nu}_{TS}$ = 151*i* cm^{-1}	27.51	27.74	70.25	26.99
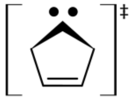 **TS**(**9**/**6**) $\bar{\nu}_{TS}$ = 414*i* cm^{-1}	10.73	10.63	68.23	10.49
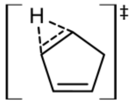 **TS**(**6**/**7**) $\bar{\nu}_{TS}$ = 551*i* cm^{-1}	2.14	2.15	68.97	1.79
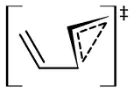 **TS**(**8**/**10**) $\bar{\nu}_{TS}$ = 173*i* cm^{-1}	29.60	30.05	72.38	28.67

[a] ωB97X-D/def2-TZVP + 0.955(E_{ZPV}) theoretical model. [b] T = 298.15 K.
